# Biochemical studies of two lytic polysaccharide monooxygenases from the white-rot fungus *Heterobasidion irregulare* and their roles in lignocellulose degradation

**DOI:** 10.1371/journal.pone.0189479

**Published:** 2017-12-11

**Authors:** Bing Liu, Åke Olson, Miao Wu, Anders Broberg, Mats Sandgren

**Affiliations:** 1 Department of Molecular Sciences, Swedish University of Agricultural Sciences, Uppsala, Sweden; 2 Department of Forest Mycology and Plant Pathology, Swedish University of Agricultural Sciences, Uppsala, Sweden; USDA Forest Service, UNITED STATES

## Abstract

Lytic polysaccharide monooxygenases (LPMO) are important redox enzymes produced by microorganisms for the degradation of recalcitrant natural polysaccharides. *Heterobasidion irregulare* is a white-rot phytopathogenic fungus that causes wood decay in conifers. The genome of this fungus encodes 10 putative Auxiliary Activity family 9 (AA9) LPMOs. We describe the first biochemical characterization of *H*. *irregulare* LPMOs through heterologous expression of two CBM-containing LPMOs from this fungus (*Hi*LPMO9H, *Hi*LPMO9I) in *Pichia pastoris*. The oxidization preferences and substrate specificities of these two enzymes were determined. The two LPMOs were shown to cleave different carbohydrate components of plant cell walls. *Hi*LPMO9H was active on cellulose and oxidized the substrate at the C1 carbon of the pyranose ring at β-1,4-glycosidic linkages, whereas *Hi*LPMO9I cleaved cellulose with strict oxidization at the C4 carbon of glucose unit at internal bonds, and also showed activity against glucomannan. We propose that the two LPMOs play different roles in the plant-cell-wall degrading system of *H*. *irregulare* for degradation of softwood and that the lignocellulose degradation mediated by this white-rot fungus may require collective efforts from multi-types of LPMOs.

## Introduction

Fungi play a critical role in decomposing plant biomass in nature [1]. Wood rotting fungi, including brown-rotting and white-rotting species, are known as efficient degraders of lignocellulosic biomass [2]. Woody biomass is mainly composed of primary and seconndaryplant cell walls, which consist of three major groups of building blocks; cellulose, hemicellulose together with pectin (primary) or lignin (secondary), respectively[3]. As the major constitution found in mature wood, the secondary cell walls have complex three-dimension structures in which the highly ordered cellulose micro-fibrils are embedded in hemicellulose, and lignin fills the space between and interconnect cellulose and hemicellulose. All three components together create a recalcitrant structure that protects plant cells from microbial degradation [3]. Brown-rot fungi act predominatly on cellulose and hemicellulose, while white-rot fungi have the capability to efficiently degrade both the lignin fraction and the wood polysaccharides [2,3]. The main difference between the cell walls of softwood (e.g. pine and spruce) and hardwood (e.g. birch, aspen and oak) is the chemical composition and structure of the hemicellulose fraction, in which softwood contains mostly glucomannan but hardwood is mostly composed of xylan [3,4].

The basidiomycete white-rot fungus *Heterobasidion annosum* (Fr.) Bref. *sensu lato* is a serious pathogen of conifer trees, which has efficient wood degradation capabilities. The economic losses caused by *H*. *annosum* s.l are at least 790 million Euros per year for European forest owners due to growth reduction and devaluation of timber at harvest [5]. *H*. *annosum* s.l causes annosus root rot in conifers in boreal and temperate forests in the northern hemisphere [6] by infecting host trees via exposed fresh wood in wounds or stump surfaces where it grows in and colonizes the wood. *H*. *annosum* s.l grows through the heartwood into the root system and can spread from one tree to another trees via root-to-root contacts [7]. *H*. *annosum* s.l is a species complex that consist of of five phylogenetically distingt species with partly overlaping geographic distrubution and host preferences [8–10]. In Europe there are three species present (*H*. *annosum sensu stricto* (s.s.), *H*. *parviporum* and *H*. *abietinum*) and in North American there are two species (*H*. *irregulare* and *H*. *occidentale*) [8–10]. *H*. *irregulare* infects and decays mostly pine trees and junipers and can grow within living trees and dominate the decomposition of wood during the competition with other fungi [5,9].

The genomes of *Heterobasidion* spp. have been shown to encode a large arsenal of putative carbohydrate active enzymes (CAZymes) and oxidative enzymes for degrading plant cell walls [11]. Lytic polysaccharide monooxygenases (LPMOs) are a class of recently described copper-dependent redox enzymes that are found both in fungi and bacteria [12]. It has been proposed that LPMOs catalyze oxidative cleavage of linkages between glycan residues in polysaccharides by employing molecular oxygen and external electron donors [13,14]. Cu(I) is used to activate O_2_ to form a Cu(II)-superoxl or a Cu(II)-oxyl reactive intermediate, and thereafter add a single oxygen either at the C1 or the C4 position of the two adjacent glycan residues at the scissile bond. The redox cycle of active-site Cu is thought to be coupled to the delivery of two electrons by an external electron donor [15]. LPMOs are generally thought to be very important components in the enzyme system of plant cell wall-degrading microorganisms for efficient cellulose degradation [16,17] due to their unique capability to introduce oxidative cleavage on the surface of crystalline cellulose [18,19]. It is generally believed that the introduction of new chain ends in the crystalline micro-fibrils by LPMOs creates new starting points for other processive cellulolytic enzymes like cellobiohydrolases [16,19]. The overall structures of LPMOs share a common β-sandwich architecture with the active site positioned on a flat surface of the molecule. The active site of LPMOs is a conserved copper-bound motif composed of two histidine residues, where one of the histidine residues is the N-terminal of the mature protein after removal of the signal peptides [20].

LPMOs have been categorized into four Auxiliary Activity (AA) families (AA9, AA10, AA11, and AA13) in the classification system of Carbohydrate-Active enZymes (CAZymes, www.cazy.org) [21,22]. Families AA9 and AA10 contain the largest numbers of unique proteins. AA9 contains only fungal LPMOs, while bacterial LPMOs are mainly found in AA10. Most of the hitherto characterized AA9 LPMOs show catalytic activity against cellulose and some of them are also active on various hemicellulosic substrates, whereas AA10 LPMOs have been found to be mostly active on chitin but some of them are also on hemicellulose [21,22]. Currently only a few LPMOs belonging to AA11 and AA13 have been identified [21,22]. These two AA families differ from AA9 and AA10 through their unique structural features or substrate specificity. AA11 LPMOs share some common structural features with both AA9 and AA10, and AA13 LPMOs are found to have activities against starch.

Genes encoding AA9 LPMOs have been identified in a wide range of different fungi, including ascomycetes and basidiomycetes, with an average number of AA9 genes at 7.4 and 8.0 respectively [21]. Among basidionmycetes, white-rot fungi have shown to have a larger number of genes encoding AA9 LPMOs (9–32) than brown-rot fungi (2–10) [21,23]. The best characterized fungal LPMO systems at present are the three ascomycetes *Neurospora crassa* (9 out of 14) [18,24–29], *Podospora anserine* (6 out of 31) [30–32] *and Myceliophthora thermophila* (4 out of 22) [33–35]. The characterization of those three ascomycete systems have shown varied substrate specificities of LPMOs on different types of cellulosic and hemicellulosic substrates, which suggests that individual LPMOs may play different rioles in both cellulose and hemicellulose degradation. Characterization of individual LPMOs in a white-rot fungal systems have not been as well addressed as the ascomycete systems so far. The only white-rot fungi with more than one LPMO chracterized so far are *Pestalotiopsis sp*. [36] and *Gloeophyllum trabeum* [37,38]. It was shown that the two LPMOs from *Pestalotiopsis sp*.(*Ps*LPMO9A and *Ps*LPMO9B) oxidize insoluble cellulose at different positions, but no information is available in terms of substrate specificity [36]. *G*. *trabeum* LPMO9A-2 (*Gt*LPMO9A-2) was shown to have oxdiative cleaving activities against both cellulose as well as different types of hemicellulosic substrates, including xyloglucan and glucomannan [37]. *G*. *trabeum* LPMO9B (*Gt*LPMO9B) was shown to act synergisticly with a endoglucanse or a xylanase on the saccharification of pretreated woody biomaterials [38], but the enzyme acitivty of *Gt*LPMO9B alone is not clear. The abundance of AA9-encoding genes in white-rot fungi suggest that white-rot fungi may also have a similar machinery of using multiple types of oxidative enzymes for liberating individual polysaccharide components from the recalcitrant structures of plant cell walls during fungal growth on woody substrates.

The *Heterobaisdion irregulare* genome contains 10 putative AA9 genes which have been named *Hi*AA9A through J [39]. Two previous transcriptomics studies have shown that five of putative AA9 genes in *H*. *irregulare* (*Hi*AA9A, *Hi*AA9B, *Hi*AA9D, *Hi*AA9H, *Hi*AA9I) were considerably up-regulated when the fungus was cultivated on pure cellulose and woody biomaterials, compared to a glucose control [11,39]. *Hi*AA9D, *Hi*AA9H and *Hi*AA9I are predicted to have a C-terminal carbohydrate binding module family 1 (CBM1) linked to their AA9 catalytic domains [39]. The CBM1 domains have been demonstrated to be strong cellulose binding modules, and the catalytic domains linked to CBM1 domains are often reported to have strong cellulose binding affinity [40]. The expression patterns of the *H*. *irregulare* AA9 genes that contain CBM1 (*Hi*AA9D, *Hi*AA9H, *Hi*AA9I) were similar to two cellobiohydrolases (CBH) in *H*. *irregulare* (*Hi*Cel7A and *Hi*Cel6A). This suggests that AA9 proteins are co-expressed with the CBHs, and therefore very likely to be involved in cellulose degradation [11]. None of the 10 LPMOs in the *H*. *irregulare* genome has been characterized to date and their enzyme activities and substrate preferences are unknown.

The aim of this study was to gain insight into the oxidative biomass-degrading enzyme system of the white-rot fungus *H*. *irregulare* by exploring the enzymatic activities of some CBM-containing LPMOs on a broad range of lignocellulosic substrates, in order to increase our knowledge of the functional diversification of LPMOs in this fungal system. Two LPMOs from *H*. *irregulare* were heterologously expressed in the methylotrophic yeast *Pichia pastoris*. The regioselectivity and substrate specificity of these two enzymes were characterized and their biochemical properties were compared with previously characterized AA9 LPMOs. The potential contributions of these two enzymes on lignocellulose degradation in the native system of *H*. *irregulare* are discussed.

## Materials and methods

### CRISPR/Cas9-mediated LPMO expression in *P*. *Pastoris*

#### Preparation of transformation plasmids

The predicted coding sequences of *Hi*LPMO9H and *Hi*LPMO9I were retrieved from the JGI *H*. *irregulare* Genome Portal (http://genome.jgi.doe.gov/Hetan1/Hetan1.home.html). Both LPMO sequences including the native secretion signal sequences were synthesized *de novo* (GeneArt ThermoFisher, Buckinghamshire, UK). All the assembly fragments were prepared by PCR with the primers designed using NEBuilder Assembly tool (http://nebuilder.neb.com/), as shown in Table 1. Transformation vectors were constructed by performing Gibson assembly with corresponding DNA fragments using NEBuilder HiFi DNA Assembly Master Mix (New England Biolabs, USA).

**Table 1:** Primers for assembly of transformation vectors.

Vector		Sequence 5′->3′
**pGAP_LPMO_zeocin plasmid**		
	*Hi*LPMO9H_Fw	TGAACAACTATTTCGAAACGATGTTTTTCACAGCCGCTC
	*Hi*LPMO9H_Rv	CTGAGATGAGTTTTTGTTCTCAAGCACTGTGAGTAATATGC
	*Hi*LPMO9I_Fw	TGAACAACTATTTCGAAACGATGTTGTTCAAGTCTTCTC
	*Hi*LPMO9I_Rv	CTGAGATGAGTTTTTGTTCTTAGGCACTGGCTATAGTAG
**HR donor plasmid**		
	pGAP_LPMO_Zeo_Fw	GCATTCTTTCCTTTCTTTTTGTAGAAATGTCTTGGTG
	pGAP_LPMO_Zeo_Rv	CAGATCCCGGTGTGGGCAAATTAAAGCCTTCGAG
	Left HR arm_Fw	CGAATTGGCGGAAGGCCGTCAAGGCCGCATGTGCCAAGAAGGTCGTCATC
	Left HR arm_Rv	ACATTTCTACAAAAAGAAAGGAAAGAATGCAATCGAG
	Right HR arm_Fw	AAGGCTTTAATTTGCCCACACCGGGATCTGGTC
	Right_HR arm_Rv	GGCAGTGAGCGGAAGGCCCATGAGGCCCAGCTATATGAAGAGTTTATTGGAGGAGCATTG
**Cas9 plasmid**		
	pGAP_promoter_Fw	CGAATTGGCGGAAGGCCGTCAAGGCCGCATTTTTTGTAGAAATGTCTTGGTG
	pGAP_promoter_Rv	GTACTTCTTGTCCATCGTTTCGAAATAGTTGTTCAATTG
	HsCas9_Fw	AACTATTTCGAAACGATGGACAAGAAGTACTCCATTG
	HsCas9_Rv	TCTTTTCTTCTTTGGGTCTCCACCGAGCTGAGA
	SV40_DAS1TT_PARS_Fw	CAGCTCGGTGGAGACCCAAAGAAGAAAAGAAAAGTTTAAAC
	SV40_DAS1TT_PARS_Rv	GGCAGTGAGCGGAAGGCCCATGAGGCCCAGTCGACAATTAATATTTACTTATTTTGG
**GPD gRNA plasmid**		
	pTEV1_StRNA2_AOX1TT _Fw	CGAATTGGCGGAAGGCCGTCAAGGCCGCATTCGAGATAAGCTGGGGGAAC
	pTEV1_StRNA2_AOX1TT _Rv	GGCAGTGAGCGGAAGGCCCATGAGGCCCAGGCACAAACGAAGGTCTCAC
**pGAPZαA backbone**		
	pGAPZαA_Fw	GAACAAAAACTCATCTCAGAAG
	pGAPZαA_Rv	CGTTTCGAAATAGTTGTTCAATTG
***pMA backbone***		
	pMA_Fw	CTGGGCCTCATGGGCCTT
	pMA_Rv	ATGCGGCCTTGACGGCCTTC

#### Homologous recombination donor plasmid

Each of the synthesized LPMO sequences was inserted upstream of the pGAP promoter in the pGAPZαA vector (Invitrogen, San Diego, CA) using PCR and Gibson Assembly with primer pairs “pGAPZαA_Fw and pGAPZαA_Rv” in combination with the primer pairs “*Hi*LPMO9H_Fw and *Hi*LPMO9H_Rv” and “*Hi*LPMO9I_Fw and *Hi*LPMO9I_Rv”, respectively. The homologous recombination (HR) donor plasmids were constructed by assembling the following three fragments into pMA vector (Invitrogen, San Diego, CA) by Gibson assembly. Fragment one was amplified from the recombinant pGAPZα vector by PCR using primer pairs “pGAP_LPMO_Zeo_Fw and pGAP_LPMO_Zeo_Rv”, which contains one expression cassette of one LPMO (*Hi*LPMO9H or *Hi*LPMO9I) with C-terminal 6x Hisditine tag and one following cassette for Zeocin resistance. Fragment two and Fragment three are homologous recombination arms with length of 991 bp to either left or right of the directed cleavage site of Cas9 enzyme, which were cloned from the genome of *Pichia pastoris* X33 strain (Invitrogen, San Diego, CA) by PCR using corresponding primer pairs “Left_HR_Arm_Fw and Left_HR_Arm_Rv” or “Right_HR_Arm_Fw and Right_HR_Arm_Rv”.

#### Plasmids expressing Cas9 and gRNA

Cas9 expression plasmid and gRNA expression plasmid were built on the basis of the constructs pGAP_HsCas9_DAS1 and pTEV1_StRNA2_AOX1TT respectively that were designed by Weninger [41]. For Cas9 expression plasmid, three fragments were assembled into pMA vector, including pGAP promoter which is amplified from pGAPZα vector (Invitrogen, San Diego, CA), encoding sequence of human (*Homo sapiens*) codon-*optimized Cas9* (*Hs*Cas9) which is cloned from p414-TEF1p-Cas9-CYC1t plasmid (Addgene plasmid no. 43802, Cambridge, USA) and a synthesized fragment (GeneArt ThermoFisher, Buckinghamshire, UK) containing SV40 signal sequence, DAS1 terminator and PASR yeast replication origin in a sequential order.

The gRNA plasmid was constructed by assembling the synthesized fragment pTEV1_StRNA2_AOX1TT (GeneArt ThermoFisher, Buckinghamshire, UK) containing the target sequence “ACCAGATCCCGGTGTGGGAA” into pMA vector (GeneArt ThermoFisher, Buckinghamshire, UK).

#### P. pastoris transformation

The preparation of competent *P*. *pastoris* cells and transformation were performed using previously described methods [41]. The HR donor plasmid was linearized by treatment of FastDigest Sfi I (Fermentas, ThermoFisher, USA) at 50°C for 20 h. 40 μl of *P*. *pastoris* X33 competent cells were mixed with 1 μg linearized HR donor plasmid, 0.5 μg Cas9 plasmid and 0.5 μg gRNA expression plasmid. Plasmids were introduced into *P*. *pastoris* cells by electroporation. Successful transformants expressing *Hi*LPMO9H and *Hi*LPMO9I were obtained after 3 days incubation at 30°C on solid yeast extract peptone dextrose (YPD; 10 g/l yeast extract, 20 g/l peptone, 20 g/l glucose) medium containing 100 μg/ml Zeocin (Invitrogen, San Diego, CA).

#### Heterologous protein expression

*P*. *pastoris* were pre-cultured in 25 ml YPD medium (25 μg/ml OF WHAT) for 20 h in a shaking incubator at 30°C, 130 rpm. 5 ml pre-culture was transferred to 1L YPD medium in a 3L-baffled flask and cultivated for 24 h at 28°C with 110 rpm orbital shaking. *P*. *pastoris* cells were removed by centrifugation at 5000 × *g* for 30 min at 25°C, and the culture supernatant was first filtered through a 0.45-μm PES membrane filter, and thereafter through a 0.2-μm PES membrane.

### Protein purification and preparation

Filtered culture supernatants containing either *Hi*LPMO9H or *Hi*LPMO9I were adjusted with ammonium sulfate ((NH_4_)_2_SO_4_) to 1 M and 1.5 M respectively in 20 mM TRIS buffer pH 7.5. The samples were applied onto a two inter-connected 5 ml HiTrap Phenyl FF HS columns (GE Healthcare Biosciences, Uppsala, Sweden), followed by a step elution with 20 mM TRIS buffer pH 7.5. The eluate was desalted using a 500 ml BioGel P-6DG column (Bio-Rad Laboratories, Richmond, USA) pre-equilibrated in 20 mM TRIS buffer pH 7.5. The retained desalted sample was purified using 5 ml Ni^2+^-charged HiTrap chelating HP column (GE Healthcare Biosciences, Uppsala, Sweden) with a step elution of 20 mM TRIS buffer pH 7.5 containing 200 mM imidazole. After buffer exchange into 20 mM BisTRIS buffer pH 6.5, the *Hi*LPMO9H sample was purified using 4.7 ml HiScreen CaptoQ ImpRes column (GE Healthcare Biosciences, Uppsala, Sweden) with a gradient from 0 to 250 mM sodium chloride (NaCl) in 50 ml of 20 mM BisTRIS buffer, pH 6.5. The *Hi*LPMO9I sample in 20 mM TRIS buffer pH 7.5 containing 1.5 M (NH_4_)_2_SO_4_ was applied onto 4.7 ml HiScreen Phenyl HP column (GE Healthcare Biosciences, Uppsala, Sweden), followed by a reverse gradient from 1.5 M to 0 M ammonium sulfate in 25 ml of 20 mM TRIS buffer pH 8.0. The fractions containing the target protein with the right molecular size, as estimated by SDS-PAGE analysis, were pooled and concentrated to 1 ml using a Vivaspin 20 concentration tube with a 10 kDa cutoff by centrifugation at 5,000 × *g* for 20 minutes. A size-exclusion chromatography step was applied as the final polishing step for both LPMOs using a 120 ml Superdex 75 column (GE Healthcare Biosciences, Uppsala, Sweden), and using 20 mM sodium acetate (NaOAc) buffer, pH 5.5 containing 150 mM NaCl, as running buffer. After Cu(II) saturation by incubation with 1 mM CuSO4 at 25°C for 1 hour, the purified protein samples were buffer exchanged into 10 mM NaOAc pH 5.5 by using pre-equilibrated SpinTrap G-25 column (GE Healthcare Biosciences, Uppsala, Sweden) with 2-min centrifugation at 800 × *g*.

Protein purity was assessed by SDS-PAGE electrophoresis on TGX Stain-Free Precast Gels with Precision Plus Protein Unstained Protein Standards (Bio-Rad Laboratories, Richmond, USA). The calculation of protein concentration was based on absorbance at 280 nm measured on a Nanodrop spectrophotometer, theoretical molecular weights and molar absorption co-efficients (*Hi*LPMO9H: 1.62; *Hi*LPMO9I: 1.37) computed using ProtParam tool on ExPASy server (http://web.expasy.org/protparam/). The N-glycosylation and O-glycosylation sites of the expressed proteins were predicted on NetNglyc server (http://www.cbs.dtu.dk/services/NetNGlyc/) and NetOGlyc server (http://www.cbs.dtu.dk/services/NetOGlyc/), respectively. The deglycosylation trials were performed using α-mannosidase from *Canavalia ensiformis* (Sigma-Aldrich, Germany) with a ratio of 1:40 (w/w) to the purified LPMO samples, and incubation at 30°C overnight, and the effects on molecular size was monitored by SDS-PAGE analysis.

### Biochemical characterization of *Hi*LPMO9H and *Hi*LPMO9I

#### Enzyme activity assays

Enzyme activity reactions were performed in 200-μl mixtures containing 1 μM enzyme (*Hi*LPMO9H or *Hi*LPMO9I), in 1 mM ascorbic acid (Asc.) and each of the following substrates: 10 mg/ml reduced phosphoric acid swollen cellulose (PASC), 0.4 mg/ml cellohexose, 2 mg/ml carboxymethyl cellulose (CMC), 2 mg/ml xyloglucan, 2 mg/ml glucomannan, 2 mg/ml barely glucan, 2 mg/ml galactomannan and 2 mg/ml glucuronoxylan, in 5 mM NaAc buffer pH 6.0 in 2 ml round-bottom microcentrifugic tubes. The substrates were mixed with one of the enyzmes or equlivant volumn of miliQ water (as control), and freshly prepared ascorbic acid was added as the last step prior to incubation. The PASC activity reactions were incubated in a thermomixer (Eppendorf, Hamburg, Germany) at 50°C for 16 hwith 1000 rpm shaking. The incubation of reaction with the other substrates was performed at 30°C for 16 h with shaking at 600 rpm for samples with polysaccharide substrates and without shaking for samples with cello-oligosaccharides. Control reactions were performed in the same conditions in absence of enzyme. Reactions were stopped by boiling the reaction samples at 95°C for 5 min. The reaction samples were thereafter centrifuged at 13,300 × *g* for 5 min. The supernatant was isolated and used to examine the generation of oxidized product. To simplify the analysis of the product profile in the following analysis, samples from reaction with glucomannan were subjected to treatment with an Endoglucanse (*Mt*EG7) from *Myceliophthora thermophile*, which was kindly provided by the research group of Prof. Paul Christakopoulos at Luleå University of Technology, Sweden. The treatment was performed by 1-h incubation at 37°C with 1 μM *Mt*EG7 in 100 μl of sample supernatant.

PASC was prepared following the protocol by Wood *et al* [42]. PASC in reduced form were prepared following the method described by Westereng *et al* [43]. Cellohexose (G6), xyloglucan from tamarind seed, konjac glucomannan (low viscosity), barely β-1,3–1,4-glucan (medium viscosity) and locus 1-4-β -D-Mannan were purchased from Megazyme (Bray, Ireland). carboxymethyl cellulose (CMC) and birchwood xylan were obtained from Sigma-Aldrich (Germany).

#### Product analysis of enzyme reaction

The supernatants of enzyme reactions were subjected to a series of -product analysis by high performance anion-exchange chromatography with pulsed amperometric detection (HPAEC-PAD), matrix-assisted laser desorption/ionization time-of-flight mass spectrometry (MALDI-TOF MS) and electrospray ionization mass spectrometry (ESI-MS/MS) as described as below.

HPAEC was performed using a CarboPac PA1 2×250 mm analytical column (Dionex Corp., Sunnyvale, CA) with eluent A (0.1 M NaOH) and B (0.1 M NaOH + 1M NaOAc) using an ICS3000 system equipped with a pulsed amperometric detectior (Dionex Corp., Sunnyvale, CA). The system was run at a constant flow rate of 0.25 ml/min at 30°C for all the sample analysis in the study. Samples from the reactions with cellulosic substrates (PASC, CMC, G6) and samples after endoglucanse treatment were analyzed using the elution gradient described by Isaksen *et al* [27]. Sample analyses from reactions with hemicellulosic substrates was following the 75-min stepwise elution method described by Agger *et al* [28].

MALDI-TOF analysis were performed following the protocol established by Vaaje-Kolstad *et al* [13]. Samples of 1 μl were mixed with 2 μl of 9 mg/ml 2,5-dihydroxybenzoic acid (DHB) in 30% acetonitrile on a MTP 384 steel target plate (Bruker Daltonics, Bremen, Germany). After dried under nitrogen flow, samples were analyzed using ULtraFlex II MALDI-TOF Mass spectrometer (Bruker Daltonics, Bremen, Germany) and data were acquired in positive-ion reflection mode.

ESI-MS/MS analysis was carried out on a Bruker maXis Impact mass spectrometer (ESI-QTOFMS) (Bruker Daltonics, Bremen, Germany) operated in positive mode scanning m/z from 50 to 3000, with calibration using sodium formate clusters. Samples of 5 μl were introduced by a HP1100 LC system (Hewlett–Packard) connected without column, using 30% acetonitrile in water (0.2% formic acid) with flow rate of 1 ml/min. Ions of *m*/*z* 683 were isolated (2 Da isolation window) and fragmented using 26.8 eV, with preset parameters for collision-induced-disassociation (100-500Da: 15-25eV, 500-1000Da: 25-30eV) All MS spectra were analyzed using the Bruker DataAnalysis 4.1 software.

#### Sequence alignment and structural comparison

Protein sequences of previously characterized AA9 LPMOs were retrieved from GenBank using the following accession numbers (*Pc*LPMO9D: BAL43430, *Nc*LPMO9F: CAD70347, *Mt*LPMO9B: AON76800, *Nc*LPMO9A: EAA30263, *Nc*LPMO9C: EAA36362, *Nc*LPMO9D: CAD21296, *Ls*LPMO9A: ALN96977). Sequences were aligned using the Mega 6.06 software (http://www.megasoftware.net/). Published LPMO crystal structures were downloaded from the Protein Database Bank (http://www.rcsb.org/pdb/home/home.do) with corresponding accession numbers (*Pc*LPMO9D: 4B5Q, *Nc*LPMO9F: 4EIR, *Nc*LPMO9A: 5FOH, *Nc*LPMO9C: 4D7V, *Nc*LPMO9D: 4EIR, *Ls*LPMO9A in complex with a cellotriose ligand: 5ACF). Previously published atomic coordinates of computational simulations of the binding of *Pc*LPMO9D onto crystalline cellulose surface was kindly provided by Dr. Gregg Beckham from National Renewable Energy Laboratory, USA. Structural comparisons and visualization were performed using PyMol molecular graphing software (DeLano Scientific LLC, San Carlos, USA).

## Results

### Heterologous expression of LPMOs from *H*. *irregulare* in *P*. *pastoris*

**Fig 1:**
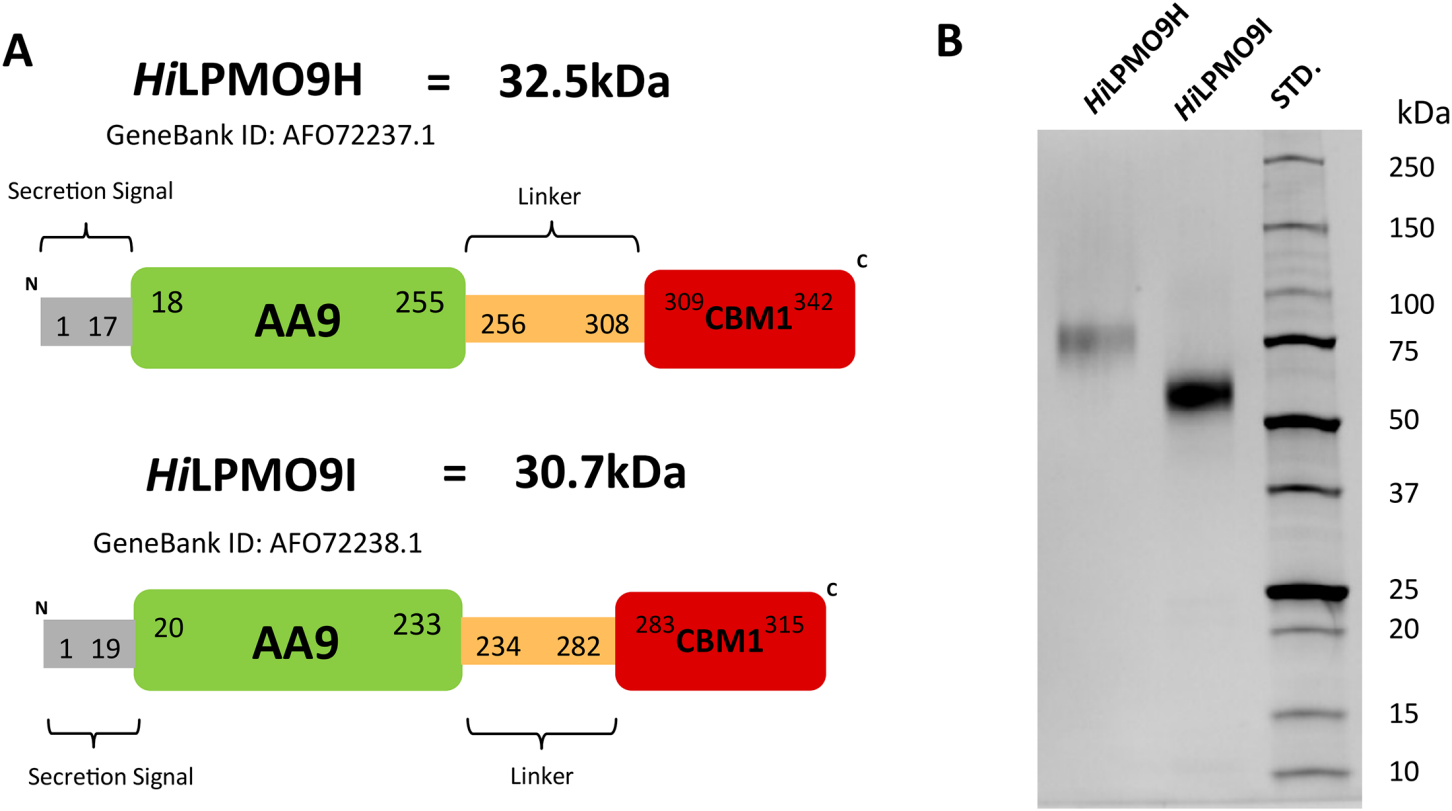
Protein properties of *Hi*LPMO9H and *Hi*LPMO9I from *H*. *irregulare*. (**A**) Schematic illustration of the modular organization of *Hi*LPMO9H and *Hi*LPMO9I, based on the prediction of conserved domain search using the translated protein sequences. The theoretical molecular weights of the mature protein were calculated while excluding the predicted secretion signal peptides. (**B**) SDS-PAGE analysis of purified proteins. Lanes from left to right are *Hi*LPMO9H, *Hi*LPMO9I and protein molecular weight standards (STD).

Two *H*. *irregulare* LPMOs that had previously showed highly increased transcription levels when the fungus was grown on woody biomass materials, *Hi*LPMO9H (GeneBank accession AFO72237) and *Hi*LPMO9I (GeneBank accession AFO72238) were successfully expressed in *P*. *pastoris* after CRISPR/Cas9-mediated gene recombination. The search of conserved domains (https://www.ncbi.nlm.nih.gov/Structure/cdd/wrpsb.cgi) revealed that both *Hi*LPMO9H and *Hi*LPMO9I contain an N-terminal AA9 catalytic domain (translated peptide sequence: 18 to 255 for *Hi*LPMO9H, 20–233 for *Hi*LPMO9I), a CBM1 domain (*Hi*LPMO9H: 309–342, *Hi*LPMO9I: 283–315) and a linker region (*Hi*LPMO9H: 256–308, *Hi*LPMO9I: 234–282) that connects the AA9 catalytic module to the CBM1domain (Fig 1A). A sequence alignment of the protein sequences of the corresponding modules of these two LPMOs reveal that the sequence similarity of the catalytic domain (45% identical) is lower than the CBM 1 domain (65% identical). *Hi*LPMO9H and *Hi*LPMO9I were predicted to have 37 and 25 *O*-linked glycosylation sites respectively, but these two LPMOs are not likely to be *N*-glycosylated due to the lack of potential *N*-glycosylation sites. The majority of the *O*-linked glycosylation sites found on *Hi*LPMO9H (62%) and *Hi*LPMO9I (64%) were predicted to occur on serine or threonine residues of the linker region. Purified of *Hi*LPMO9H and *Hi*LPMO9I were visible as protein bands of 75 kDa and 55 kDa, respectively, when resolved by SDS-PAGE (Fig 1B). Deglycosylation with α-mannosidase was performed to remove potential *O*-linked glycosyl moieties from the LPMOs expressed in *P*. *pastoris*. However, no significant reduction in protein mass was observed. The shapes of the two protein bands were slightly broadened, and the observed sizes were larger than the corresponding theoretical molecular weights for the two proteins, 32.5 kDa and 30.7 kDa, respectively (Fig 1A). This difference might be caused by the presence of multiple types of O-linked glycosylationas the result of heterologous protein expression in *P*. *pastoris*.

### Enzyme activity against cellulose

**Fig 2:**
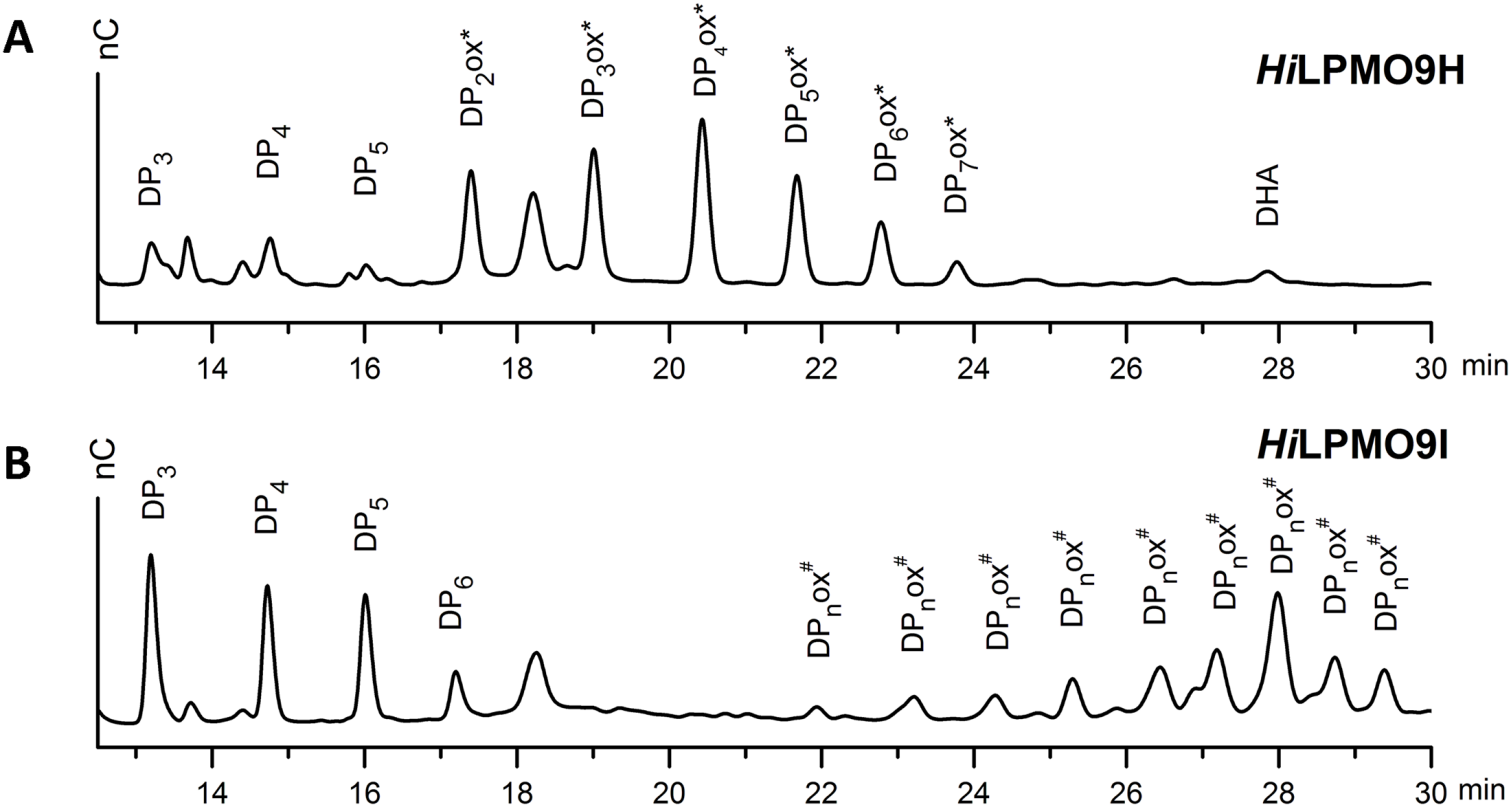
HPAEC-PAD (high performance anion-exchange chromatography with pulsed amperometric detection) analyses of the glucan products after treating PASC with two different *Hi*LPMOs using ascorbic acid as the electron donor. (**A**) HAPEC-PAD elution profile of reaction products of PASC with 1 μM *Hi*LPMO9H in the presence of 1 mM ascorbic acid. DP_n_ stands for native cello-oligosaccharide. DP_n_ ox* represents C1-oxdized aldonic sugars (DP = 2 to 6). DHA stands for dehydroascobic acid. (**B**) HPAEC-PAD elution profile of reaction products when treating PASC with 1 μM *Hi*LPMO9I in the presence of 1mM ascorbic acid. C4-oxidized products are labeled as DP _n_ ox^#^ [27].

Native and oxidized cello-oligosaccharides were identified from the reactions of *Hi*LPMO9H or *Hi*LPMO9I on PASC using 1 mM ascorbic acid as the electron donor by high-performance anion exchange chromatography (HPAEC) with pulsed amperometric detection (PAD) (Fig 2). The products generated by *Hi*LPMO9H were detected as an array of native cello-oligosaccharides with degree of polymerization (DP) from 3 to 5 as well as C1-oxidized cello-oligosaccharides (DP2—DP7), as shown in Fig 2A. The C1-oxidized products of *Hi*LPMO9H were eluted after the native sugars at retention times ranging from 17.4 min to 23.7 min. Native cello-oligosaccharides (DP3 to DP6) as well as C4-oxidized products were detected when *Hi*LPMO9I acted on PASC in the presence of ascorbic acid. C4-oxidized cello-oligosaccharides were detected as elution peaks between 21.9 min and 29.4 min (Fig 2B), and similar results have been shown in previous studies on other LPMOs strictly oxidizing at C4, e.g. *Nc*LPMO9C [27] and *Mt*LPMO9C [34]. No overlapping peaks were found between the chromatographic regions of C1-oxidized products of *Hi*LPMO9H and C4-oxidized products of *Hi*LPMO9I (Fig 2), indicating that *Hi*LPMO9H is strictly active at the C1 position of β-1,4-glycosidic linkages of PASC whereas *Hi*LPMO9I performs the oxidation only at the C4 position.

**Fig 3:**
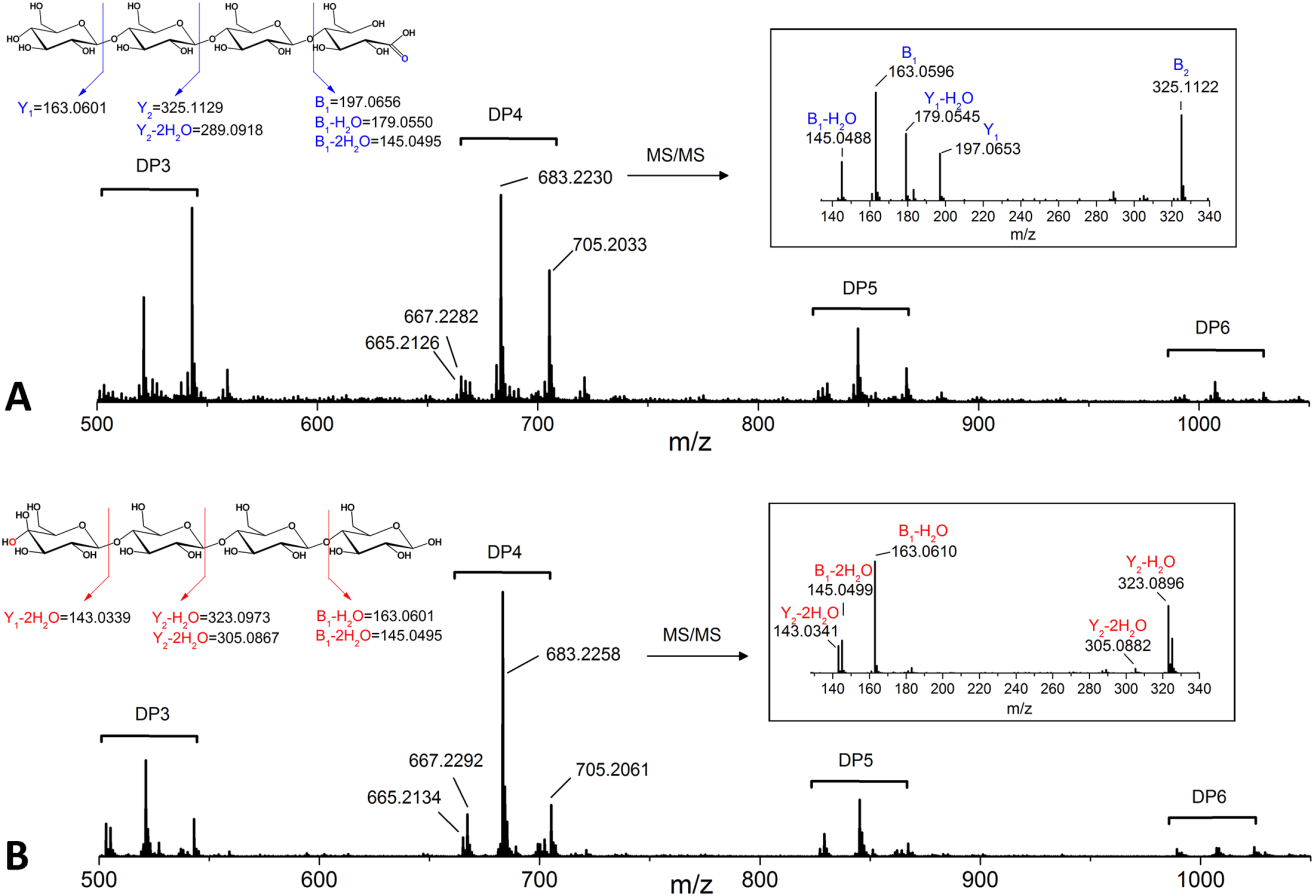
ESI-MS/MS (electrospray ionization tandom mass spectrometry) analyses of the products released by two different *Hi*LPMOs when incubated with PASC in the presence of 1mM ascorbic acid. (**A**) Bottom: mass spectra of cello-oligosaccharides produced by *Hi*LPMO9H; Top left: schematic fragmentation pattern of C1-oxidized aldonic cellotetraose and resulting product ions, and both of the precursor ion and fragment ions are proton adducts; Top right: MS/MS spectra of oxidized product with m/z 683.2230. (**B**) Bottom: mass spectrum of cello-oligosaccharides produced by *Hi*LPMO9I; Top Left: schematic fragmentation pattern of C4-oxidized gemdiol cellotetraose and the resulting product ions, and both of the precursor ion and fragment ions are proton adducts; Top right: MS/MS spectra of oxidized product with m/z 683.2258.

To further study the formation of oxidized cello-oligosaccharides released from PASC by *Hi*LPMO9H and *Hi*LPMO9I, the hydrolysates were analyzed by electrospray ionization mass spectrometry (ESI-MS). The different cello-oligosaccharides (non-oxidized and oxidized forms) were detected as an array of positive ion clusters with 1+ charge ranging from DP3 to DP6 (Fig 3). Each cluster contained four types of ions, which correspond to proton adducts of non-oxidized cello-oligosaccharides (mass-to-charge ratios (m/z) = 505, 667, 829, 989; DP3 to DP6), proton adducts of oxidized cello-oligosaccharides (C1-lactone or C4-ketone: m/z 503, 665, 827, 987; C1-aldonic acid or C4-gemdiol: m/z 521, 683, 845, 1005; DP3 to DP6), and sodium adducts of the C1-aldonic acids or C4-gemdiols (m/z +22Da, compared to the corresponding proton adduct). The ions of the highest relative intensity among all the cello-oligosaccharide products in these two reactions were the proton adducts of cellotetraose as C1-aldonic acid (m/z 683.2230 for *Hi*LPM9H) and C4-gemdiol (m/z 683.2258 for *Hi*LPMO9I), respectively (Fig 3). These ions were selected for collision-induced fragmentation to further verify the position of oxidization. The MS/MS spectra in both cases showed that the major product ions resulted from breaking glycosidic linkages (Fig 3). For *Hi*LPMO9H, fragmentation on the ion with m/z 683.2230 generated two product ions (m/z = 197.0653 and 179.0545) in good agreement with an Y_1_ fragment ion of aldonic acid cellotetraose, and its corresponding ion after elimination of water (Y_1_-H_2_O). Fragmentation of the ion with m/z 683.2258 from the reaction of *Hi*LPMO9I, gave two fragment ions (m/z = 143.0341 and 323.0896), which are in line with B_1_-2H_2_O and Y_2_-H_2_O, respectively, for a C4-gemdiol cellotetraose.

### Enzyme activity against hemicellulose

**Fig 4:**
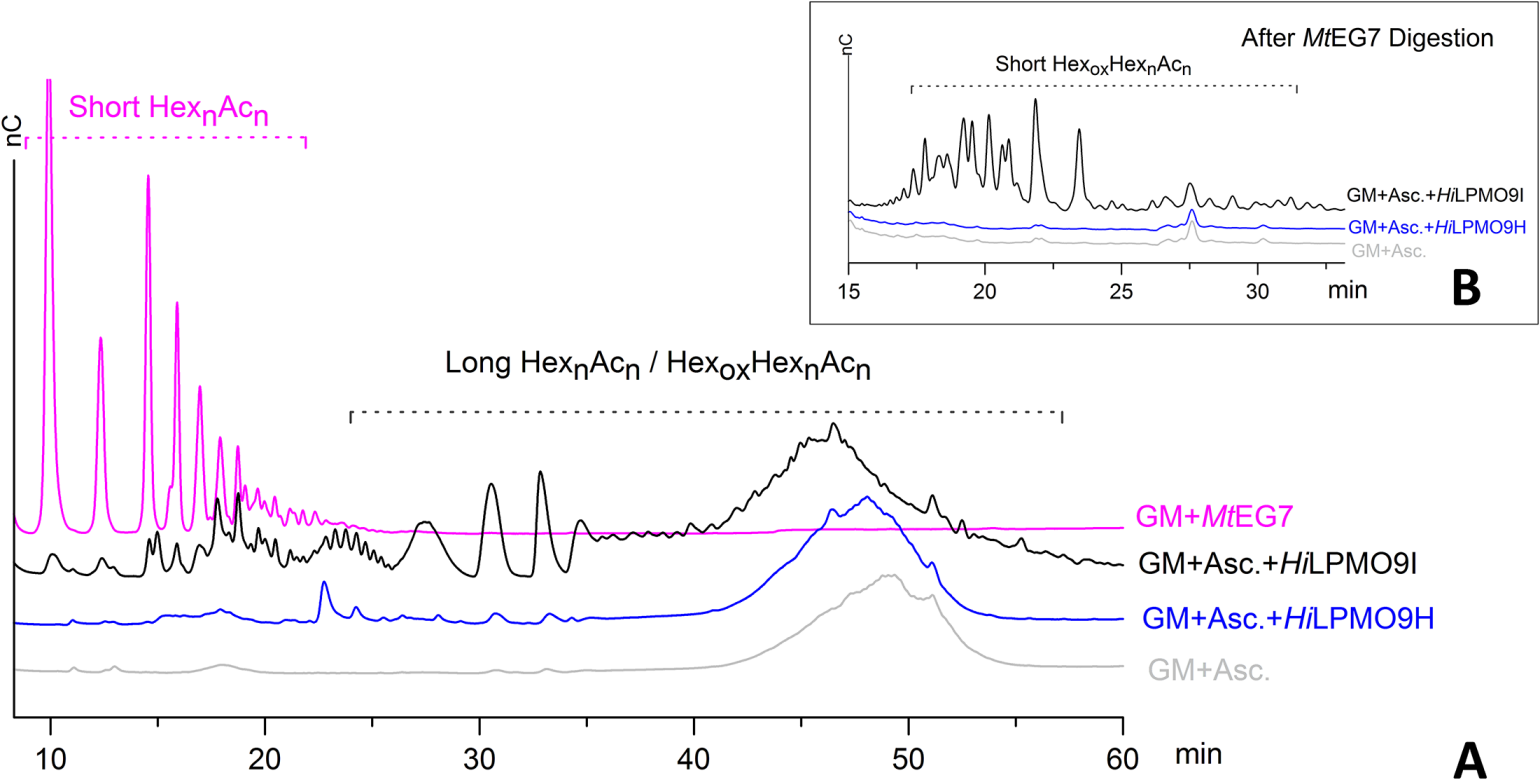
HPAEC-PAD elution profile of glucomanan samples after incubation with different types of enzymes. (**A**) when incubated with 1mM ascorbic acid alone (light grey), *Hi*LPMO9H and 1mM ascorbic acid (blue), *Hi*LPMO9I and 1mM ascorbic acid (blue) or *Mt*EG7 alone (Pink). The abbreviation “Hex” stands for the backbone hexose (glucose or mannose) and “Ac” represents the potential substitution of acetyl groups on C6 of hexose. (**B**) HPAEC-PAD elution profile of the samples after further depolymerization using *P*. *pastoris*-expressed *Mt*EG7. All the samples were incubated with 1μM *Mt*EG7 at 37°C for 1 h prior to HPAEC-PAD analysis. The HPAEC-PAD analysis was performed using a shorter elution gradient (45-min) for better visualization of peaks representing short oxidized glucomannan residues. The main difference among the elution profiles are mainly found between 15 min to 30 min. Short native glucomannan eluted before 15 min are not shown), in which no difference in elution pattern were found among three individual runs.

Samples from the incubation of konjac glucomannan (GM) with LPMOs in the presence of ascorbic acid and samples treated only with an endoglucanase (no ascorbic acid) were analyzed using a 75-min gradient HPAEC protocol established by Agger *et al* [28]. The elution pattern of the control reaction (only GM and ascorbic acid) showed a main peak eluted from 40 to 55 min (with the peak center at 49 min), which represented the intact form of the glucomannan polymers in the absence of enzyme. The sample after the treatment with *M*. *thermophile* endoglucanse 7 (*Mt*EG7) (without ascorbic acid added) showed multiple peaks in early stage (9.9 min to 22 min) of the gradient elution and there was no PAD response afterwards, which suggested that the substrate had been degraded into the oligosaccharides with low degree of polymerization. When treating the glucomannan substrate with *Hi*LPMO9I in the presence of ascorbic acid, a reduction of polymer size of glucomannan was observed (Fig 4A). The polymer peak center was shifted by 5 min towards an earlier retention time than that of intact polymer in the control sample, and a range of smaller-size oligosaccharides were detected before the main polymer peak (Fig 4A). After further digesting all the reaction products with *Mt*EG7 and analyzing the products using a shorter HPAEC elution protocol (45 min), a broad range of small peaks (16–34 min retention time) were only observed in the glucomannan sample from the reaction with *Hi*LPMO9I after the glucomannan oligosaccharide were eluted (5–14 min; Fig 4B). The additional small peaks were likely oxidized glucomannan oligosaccharides (Fig 4B). When glucomannan was treated with *Hi*LPMO9H using ascorbic acid as electron donor, a small shift of the polymer peak center (0.5 min earlier in retention time) was observed and some positive but weak PAD responses were shown in the oligomer regions, compared to the chromatogram of the control, (Fig 4A). However, after further depolymerization using *Mt*EG7 there was no difference in elution pattern observed between the sample from *Hi*LPMO9H and the control sample, suggesting that no oxidative cleavage had occurred on the glucomannan substrate during the incubation with *Hi*LMO9H in the presence of ascorbic acid (Fig 4B).

**Fig 5:**
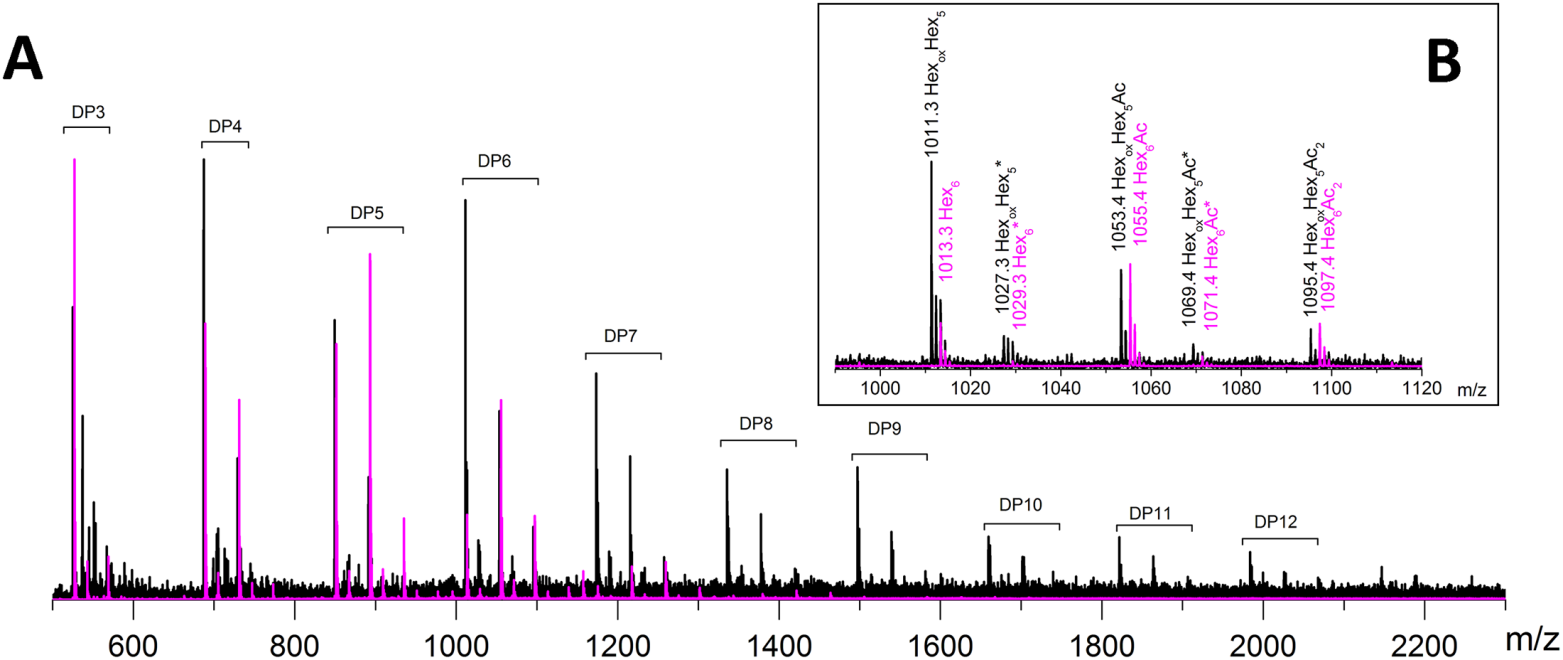
MALDI-TOF MS (matrix assisted laser desorption ionisation time-of-flight mass spectrometry) analysis for comparing the product profiles of *Mt*EG7 expressed in *P*. *pastoris* and *Hi*LPMO9I expressed in *P*. *pastoris* after incubation with glucomannan. (**A**) MALDI-TOF MS spectrum of reaction products after incubation with *Mt*EG7 alone (pink), and with *Hi*LPMO9I in the presence of ascorbic acid (black). (**B**) Comparative view of products at DP6 generated by *Mt*EG7 (pink) or *Hi*LPMO9I (black). Oxidized glucomannan oligosaccharides were detected in the spectra of *Hi*LPMO9I (black), represented as ions with 2 Da less than the native products (pink). Ions labeled with start mark (*) are potassium adducts. Ions without special mark are sodium adducts.

Matrix-assisted laser desorption/ionization time-of-flight mass spectrometry (MALDI-TOF MS) confirmed that oxidized products were generated from glucomannan by *Hi*LPMO9I using ascorbic acid as electron donor (Fig 5A). MALDI-TOF MS analysis showed that a wide range of native and oxidized oligosaccharides were generated (DP3 to DP13) after treated with *Hi*LPMO9I, whereas only native glucomannan oligosaccharides were detected in the sample after incubation with *Mt*EG7 (DP3 to DP8) (Fig 5A). The difference of the products from the separate treatment of *Hi*LPMO9I and *Mt*EG7 are illustrated by comparing the product profiles at DP6 (Fig 5B). In both cases the main products were sodium adducts of native oligosaccharides, with or without substitution by acetyl groups, which were represented by ions with m/z 1013.3 (n = 6), 1055.4 (n = 6, +1Ac) 1097.3 (n = 6, +2Ac). A small proportion of potassium adducts of corresponding native oligosaccharides (m/z 1029.3 and 1071.4) were also detected. Besides native glucomannan oligosaccharides, oxidized oligosaccharides were produced by *Hi*LPMO9I, which were present as ions sodium adducts with m/z 1011.3, 1053.4 and 1097.3 or potassium adducts with m/z 1027.3 and 1069.4.

### Regio-selectivity and substrate specificity

*Hi*LPMO9H and *Hi*LPMO9I, were incubated separately with a range of cellulosic and hemicellulosic substrates to determine the substrate specificity, and the generation of oxidized product was examined by HPAEC-PAD and mass spectrometric analysis. The results from these studies are summarized in Table 2. *Hi*LPMO9H showed oxidative cleaving activity against insoluble cellulose (PASC), but not on soluble cellulose derivative CMC or on short cello-oligosaccharides (G6). For *Hi*LPMO9H, no activity was found on the hemicellulosic substrates tested. *Hi*LPMO9I was active on PASC, but no activity was observed against other single-chain cellulosic substrates (CMC and G6). *Hi*LPMO9I could oxidatively cleave konjac plant glucomannan with heterogeneous backbone composed of glucose and mannose, whereas it could not cleave locus bean gum galactomannan with β-1,4-linked mannose backbone. This suggested that the oxidative cleavage occurred on the β-1,4-glycosidic linkage of glucomannan. No activity against other hemicellulosic substrates was detected for *Hi*LPMO9I, including tamarind xyloglucan, barley β-1,3–1,4-glucan and birchwood xylan.

**Table 2:** Regio-selectivity and substrate specificity of *Hi*LPMO9H and *Hi*LPMO9I.

	*Hi*LPMO9B	*Hi*LPMO9I
**Regio-selectivity**	C1	C4
**Cellulosic substrate**		
PASC	**+**	**+**
CMC	**-**	**-**
Cellohexose	**-**	**-**
**Hemicellulosic substrate**		
Xyloglucan	**-**	**-**
Glucomanan	**-**	**+**
β-1,3–1,4-glucan	**-**	**-**
β-Galactomannan	**-**	**-**
Glucuronoxylan	**-**	**-**

#### Sequence alignment and structural comparison

**Fig 6:**
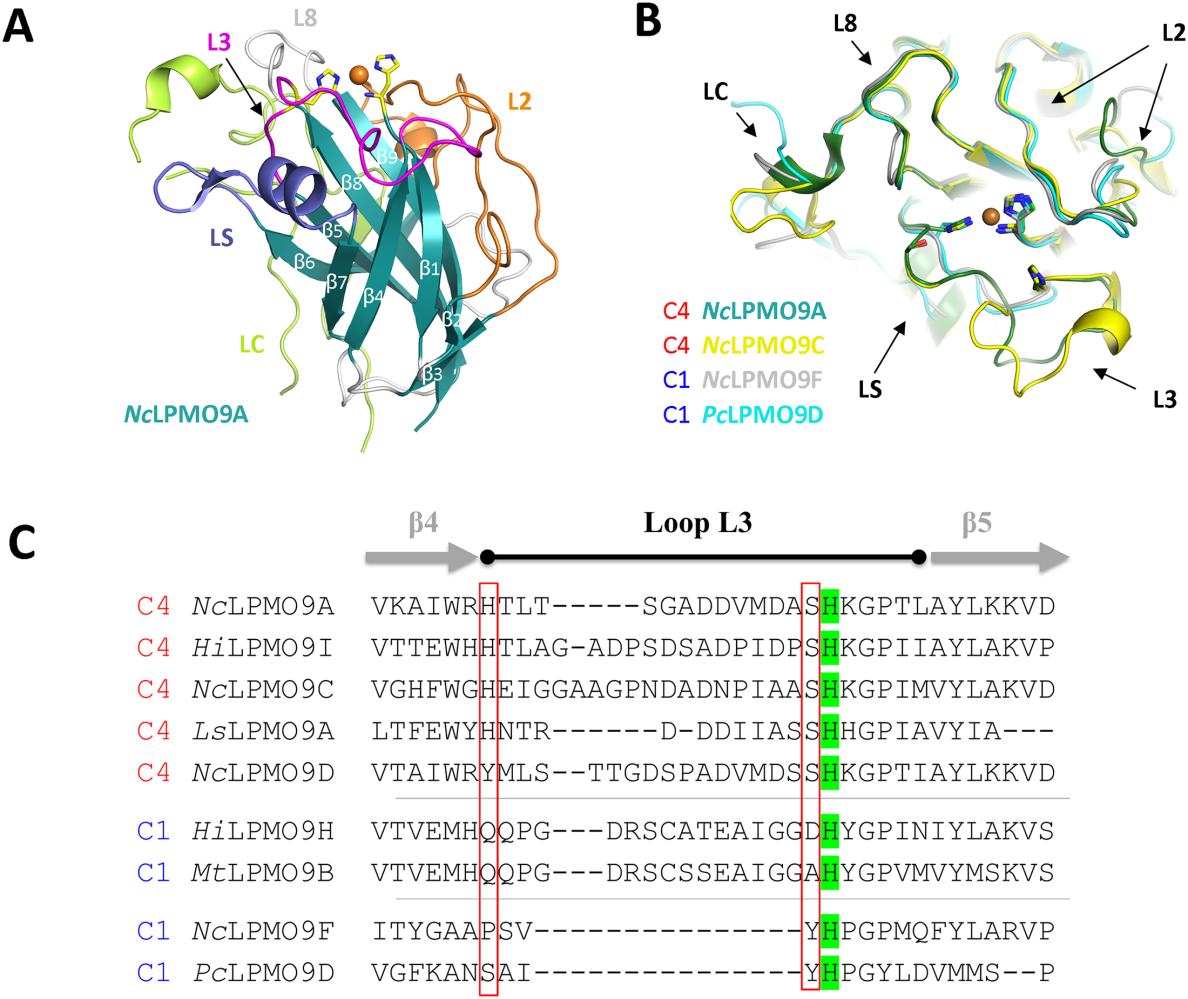
Structural features of C4-specific LPMOs and sequence alignment of C1-specific and C4-specific LPMOs. (**A**) Overall structure of a C4-specific LPMO, *Nc*LPMO9A (PDB ID: 5FOH). (**B**) Close-up view of the potential substrate binding surface of C1-specific and C4-specific LPMO structures (*Nc*LPMO9A, PDB ID: 5FOH; *Nc*LPMO9C, PDB ID: 4D7V; *Pc*LPMO9D: 4B5Q, *Nc*LPMO9F: 4EIR). (**C**) Highlights on the L3 loop regions from the sequence alignment of the biochemically characterized LPMOs that oxidize either at C1 or C4. The secondary structure information of *Nc*LPMO9A is labeled on the top of the sequences. The assigned secondary structure is a shared common feature in all structures with different numeration in the corresponding β sheets. The second active-site histidine residue is highlighted in green background.

The structural overlay (Fig 6A) showed that the major differences on the potential substrate binding surface between C1-specific and C4-specific AA9 LPMOs were observed on the L3 loop (between β5 and β6), where the second conserved histidine comprising the Cu-binding site is located (Fig 6A). A relatively short L3 loop (around 10 residues) has been observed in the structures of two C1-active LPMOs (*Pc*LPMO9D and *Nc*LPMO9F) (Fig 6B). In contrast, the L3 loop of two C4-specific LPMO (*Nc*LPMO9A and *Nc*LPMO9C) are found to be extended to 19 and 23 amino acid residues, respectively (Fig 6B and 6C). A multiple sequence alignment of all known C4-specfic LPMOs, including the C4-oxdizing *Hi*LPMO9I in our studies, reveal that the first residue of the L3 loop is an aromatic residue (histidine or tyrosine), and the residue right before the second histidine residue coordinating the catalytic cupper in the active site is found to be a serine in all included C4-specific enzyme sequences (Fig 6C). A structural overlay shows that side-chain conformations of the two conserved residues vary less compared to the other parts of the L3 loop (Fig 6B). The alignment also shows that the two C1 LPMOs, *Hi*LPMO9H and *Mt*LPMO9B also has relatively long L3 loops compared to the other two C1-specific LPMOs included in the sequence alignment (Fig 6C). However, it was found in the sequences of *Hi*LPMO9H and *Mt*LPMO9B that a glutamine residue aligns to the first conserved aromatic residue and the residues before the second active-site histidine residue is instead an aspartic acid or alanine (Fig 6C).

## Discussion

The abundance of putative carbohydrate degrading enzymes identified by a genomic sequencing approach reveal that the white rot fungus *H*. *irregulare* possess an extensive enzymatic machinery for degradation of complex plant polysaccharides [11,23]. Previously published transcriptomic studies suggested that the putative enzymes encoded by genes that were up-regulated when the fungus was grown on woody biomaterials might be employed for the depolymerization of lignocellulosic substrates in natural environment [11,39]. In this study, two of *H*. *irregulare*’s CBM-containing AA9 proteins (*Hi*AA9H, *Hi*AA9I) were confirmed to be oxidative cellulolytic enzymes. The CBM1 domain of these two LPMOs have high sequence similarity, and both are predicted to be cellulose binding modules. These two LPMOs were found to have different regio-selectivities when catalyzing oxidative cleavage of insoluble cellulose. *Hi*LPMO9H specifically oxidized at the C1 position of the pynanose rings on the β-1,4-glycosidic linkages, whereas *Hi*LPMO9I specifically oxidized at the C4 position of the glucose units on the internal bonds.

Despite the abundance of putative AA9-endcoding genes in fungal genomes only a few AA9 enzymes have been biochemically characterized to date. In addition to the two AA9 LPMOs characterized in this study, only 26 other AA9 LPMOs have previously been characterized in terms of regio-selectivity and substrate specificity, among the 1803 putative AA9 LPMOs detected in 218 sequenced fungal genomes currently available [44]. The correlation between substrate specificity of LPMO and their potential contribution on plant-polysaccharide degradation possessed by fungi has been discussed in several previous studies [28,32,34]. For C1-specific LPMOs only cellulolytic activity has been reported [18,31,32,34,43], whereas C4-specific LPMOs and LPMOs oxidizing at both the C1 and the C4 position (C1/C4-active LPMO) show broader substrate specificities with activities on hemicellulose and/or soluble cellulosic fractions, in addition to insoluble cellulose [27,28,32,37,45]. Similarly, the C1-specific *Hi*LPMO9H characterized in this study only has activity against PASC, among the range of cellulosic and hemicellulosic substrates tested. The C4-specific *Hi*LPMO9I has a wider substrate specificity and has activity also against both insoluble cellulose and glucomannan.

Plant cell wall hemicellulose composition varies widely between different plant species, tissue type as well as the developmental stage of the plant. The vast majority of LPMOs with reported hemicellulolytic activities have been shown to be active on xyloglucan, which is the main component of the primary plant cell wall [28,32]. Xyloglucan-active LPMOs either cleave strictly at the β-1,4-glycosidic bonds without xylose substitution [28,32], or randomly cleave the xyloglucan backbone regardless of the side-chain substitution [28,32]. However, the conventional woody biomaterials contain mainly secondary cell walls, in which xylan and glucomannan are the dominant compounds in hemicellulose fractions of both hardwood and softwood [4]. To the date, only a few studies have reported LPMOs that display activity against various xylan or glucomannan structures. Oxidative cleaving activity against xylan has so far only been reported for three LPMOs from *Myceliophthora thermophila* [33,34], and the xylan cleaving activities were only detected when testing these enzymes on glucuronoxylan substrates in complex with amorphous cellulose. Two of the *M*. *thermophila* LPMOsalso show activities against xyloglucan and various mixed-linkage β-glucan [33,34], but no activity against glucomannan has been reported among these enzymes [33,34]. With exception to the present study, only three studies have reported LPMOs that display activity against glucomannan so far [28,37,46]. One such case is the *N*. *crassa* C4-specific LPMO *Nc*LPMO9C, which cleaves glucomannan and several other hemicellulosic substrates containing β-1,4-glycosidic bonds, including xyloglucan and β -1,3–1,4-linked glucan from barley and lichen [27,28]. *Nc*LPMO9C has been shown to only cleave the substrate at the main-chain glycosidic linkages where the glucose units are not decorated with other sugar moieties, when the enzyme was tested on short xyloglucan and β -1,3–1,4-linked glucan oligosaccharides. This suggests that *Nc*LPMO9C probably only acts on glycosidic linkages without acetylation of the glucomman substrates. However, this hypothesis is yet not experimentally verified. The second example is the C1/C4-active *Gt*LPMO9A-2, which also shows a relatively weak glucomannan cleaving activity compared to its activity against xyloglucan. This was demonstrated by the ability of this enzyme to reduce the viscosity of the substrate in the presence of dithiothreitol [37]. Compared to *Nc*LPMO9C, *Gt*LPMO9A-2 cleaves at any β-linkage of xyloglucan backbone regardless of the xylose substitution. The authors of the study proposed that *Gt*LPMO9A-2 most likely is employed for degradation of xyloglucan in the enzymatic system of this brown-rot fungus. A recent study by Simmons and coworkers has shown that *Ls*LPMO9A and *Cv*LPMO9A are able to oxidatively cleave glucosyl-1,4-mannosyl linkages on the backbone of glucomannan, but these two LPMOs have also been shown to have activities against other hemicellulose polysaccharides [46].In the present study, it was shown that *Hi*LPMO9I only displays activity against glucomannan and not against other β-linked hemicellulosic substrates, and therefore possesses a stricter substrate preference than the other currently known glucomannan-active LPMOs. The strict glucomannan cleaving preference of *Hi*LPMO9I suggests that this enzyme is less likely to act only on the non-substituted glycosidic bonds in the manner of *Nc*LPMO9C. There might therefore be some important underlying structural features that contribute to its strict specificity against glucomannan. From a biological perspective, this would also suggest that *Hi*LPMO9I is an important component in the hemicellulolytic enzyme systems of *H*. *irregulare*, by specifically cleaving glucomman by an oxidative mechanism during the fungus decomposition of the hemicellulose fractions of softwood.

Cellulose microfibrils in plant cell walls are covered by a sheath of hemicellulose polysaccharides, such as xylan, glucuronoxylan, arabinoxylan, glucomannan, and xyloglucan. [3]. The hemicellulose polysaccharides that are wrapped over the cellulose microfibrils act as protective barriers against the cellulolytic-specific enzymes by preventing these enzymes from accessing and depolymerizing the cellulose. It is known that fungi also secrete a range of hemicellulases such as β -glucanases, xylanases and mannanases in order to break up the hemicellulose wrapping and thereby increase the accessibility of the substrate for cellulases [47]. A previous study tested the activity of an LPMO against a PASC-hemicellulose complex substrate and it was shown that a LPMO without xylogluan cleaving activity (*Nc*LPMO9A from *N*. *crassa*) could not generate oxidized cello-oligo products when incubated with PASC due to the protection by xyloglucan. However, oxidative cleavage activity against PASC was detected after the removal the xyloglucan shield by using a xyloglucanases prior treating this substrate with the LPMO [45]. The activities of *Hi*LPMO9I against both insoluble cellulose and glucomannan suggest that this enzyme could play multiple roles in the degradation process of both the hemicellulose and cellulose fractions of softwood, and thereby increasing the accessibility of the substrate for other cellulose-specific LPMOs, e.g. *Hi*LPMO9H. The proposed synergy between different enzyme types could be a part of a well-orchestrated enzyme machinery that increases the utilization efficiency of available carbon sources for the growth of *H*. *irregulare* on wood.

**Fig 7:**
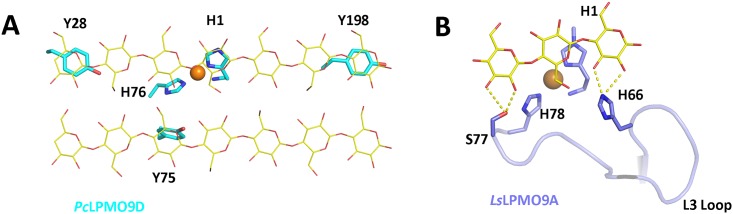
Different types of proposed LPMO-substrate interactions and comparative view on structural features of flat substrate-binding surface of LPMOs. (**A**) Superposition of the flat surface of C1-specific *Pc*LPMO9D structure (PDB ID: 4B5Q) onto a modeled cellulose surface. (**B)** Protein-ligand interaction of the C4-specific *L*. *similis Ls*LPMO9A (expressed in *Aspergillus oryzae*) in complex with cellotriose with the L3 loop indicated (PDB ID: 5ACF).

Much effort has been made to investigate the interactions between LPMOs and their potential substrates in order to understand the structural basis of the regio-selectivity that individual AA9 LPMOs possess, which have been shown biochemically [22,48,49]. The positioning of the intermediate species (Cu(II)-superoxyl or Cu(II)-oxyl) in the active site of the LPMO is thought to have a major impact on the selectivity of the oxidation of the glycan, and the way that LPMO interacts with the substrate is regarded as one of the key determinant factors for the catalytic reaction mechanism of this class of enzymes [48,50]. Recent studies have shown that the C1-specific and C4-specific AA9 LPMOs may interact with cellulose substrate in different ways. Computational simulation studies with a C1-specific LPMO from *Phanerochaete chrysosporium* (*Pc*LPMO9D) showed that the characterized LPMO could be absorbed onto a crystalline cellulose surface via hydrophobic interaction by positioning three tyrosine residues (Y28, Y75 and Y198) on the flat protein surface over glucose moieties in the cellulose chains (Fig 7A) [48]. A recent study [49] showed that a C4-specific LPMO from *Lentinus similis* (*Ls*LPMO9A) could interact with a cello-oligo ligand (cellotriose (PDB ID: 5CAF) or cellohexose (PDB ID: 5CAI)) via hydrogen-bonding. The residues His66 and Ser77 located on the L3 loop of *L*. *similis Ls*LPMO9A were involved in hydrogen bonding interactions with the substrate in both cases (Fig 7B). Interestingly, the L3 loop of AA9 LPMOs is the most variable region on the potential substrate binding surface of these enzymes, with respect to both the length and the conformation when comparing different subclasses of LPMOs (Fig 6B and 6C). Multiple sequence alignment suggests that a long L3 loop region is observed in both C1-sepcific and C4- specific LPMOs, but C4-specific LPMOs can be distinguished from C1-specific LPMOs if there is an aromatic residue (histidine or tyrosine) as a first residue in the L3 loop region and a serine residue right before the second histidine residue coordinating the catalytic copper in the active site (Fig 6C). The presence of two additional conserved residues on the L3 loop that are specifically observed among C4-specific LPMOs (Fig 6C), and their potential potency of interaction with target substrate via hydrogen bonding (Fig 7B), suggest that there might be some correlation between those features and their strict C4 oxidizing activity.

In summary, this study has characterized two out of the ten putative AA9 LPMOs from *H*. *irregulare*; *Hi*LPMO9H and *Hi*LPMO9I, and this is the first biochemical characterization study of the oxidative biomass-degrading enzyme system of this white-rot fungus. These two LPMOs cleave the glycosidic linkages in cellulose by oxidizing different carbon positions of the glycan. The difference in oxidization preference on cellulose between the two LPMOs suggests that this fungus most likely utilize more than one of its set of LPMOs for the degradation of cellulose. Further biochemical studies of *Hi*LPMO9H and *Hi*LPMO9I, with the focus on comparing the actions of these two enzymes on cellulose surface, could provide more insight regarding the diversity of different types of LPMOs on cellulose degradation. *Hi*LPMO9H only displayed activity against insoluble cellulose, whereas *Hi*LPMO9I possessed a broader substrate specificity with activity against both cellulose and glucomannan. The different substrate specificities indicate that these two *H*. *irregulare* LPMOs might both play important but different roles in plant-cell-wall depolymerization during degradation of softwood biomass by this fungus. The development of methods for precise genome editing in *H*. *irregulare* would enable gene knock-out experiments to confirm the proposed roles of *Hi*LPMOs in lignocellulosic degradation.
